# Supplementary material for: Learning curve and surgical outcome of robotic assisted colorectal surgery with ERAS program
Source: Sci Rep. 2022 Nov 29;12:20566. doi: 10.1038/s41598-022-24665-w (PMC9709162; doi:10.1038/s41598-022-24665-w)
Supplement: Supplementary file 2 — Supplementary Information 2. [file 41598_2022_24665_MOESM2_ESM.zip › RAL Raw data-Ñ[▒K/120/HS_ñw╝╨░O▒Kñσ.pdf]

正式摘要(電子簽章：000000228070144)

W52 / W52 ☐ 英文標題(注意:無英文姓名-請補病人基本檔!) ☐【讀取】憑證

臺中榮民總醫院

出院病歷摘要

轉入號： 轉出號：

[redacted]

(8)流水編號	
(9)入院日期	110/10/20 (2021) 直腸外科 W52 - 112
(10)轉科日期	
(11)出院日期	110/10/25 (2021) 住院天數計 5 日

診斷

- 入院 :Rectal cancer, cT4N1bMx
- 出院 :Rectal cancer, cT4N1bMx, s/p Robotic LAR on 2021/10/21

主訴

- bloody stool for about 2 months

病史

- This 48 years old male patient has past history of BPH s/p laser TURP on 2021/8. He complained bloody stool for about 2 months and constipation recently. He came to LMD for help. A partial obstructed tumor at 15cm FAV was found and biopsy. Malignancy was suspected. He was transferred to our hospital. Abdominal CT showed sigmoid-rectal tumor sized 6.5cm and invaded to visceral peritoneum or adjacent organs with two or three regional lymph nodes. Under the impression of rectal cancer, he was admitted for surgical intervention.

體檢發現

- Anal inspection:
  Recto digital examination:
  Sigmoidoscopy:
  Anoscopy:
  Abdomen:
  - General appearance :
    - easy looking , conscious clear , without cardiopulmonary distress
    - Vital signs自動帶入date 體溫(BT) 脈搏(P) 呼吸(R) 血壓(BP) 身高(BH) 體重(BW) BMI
      20211020 35.7℃ 83次/分 18次/分 111/73mmHg 168.1cm 71kg 25.1
  - HEENT :
    - Eyes: no anemic conjunctiva, no icteric sclera, normal EOM
    - ENT: no otorrhea, no rhinorrhea, no erythematous mucosa, no injected tonsil, no deformity
  - Neck: supple, freely movable, trachea midline,no deformity, no engorged jugular vein , no palpable thyroid or lymph node enlargement
  - Chest:
    - Inspection: Grossly normal, symmetric and fully expansion, no accessory muscle used, no operation scar, no spider angiomata, no gynecomastia
    - Palpation: no palpable mass or nodule, no subcutaneous emphysema, no tenderness
    - Ausculation: clear breathing sound
  - Heart:
    - Percussion: normal heart size
    - Ausculation: regular heart beat, normal S1, S2, no gallop, no murmur
  - Abdomen:
    - Inspection: in
      - flat contour, symetrically, no scar, no stria, no dilated veins, no rashes
    - Ausculation: normoactive bowel sound, no abdominal bruits
    - palpation: soft, no muscle guarding or rigidity, no local tenderness, no rebounding pain, no palpable mass, no palpable pulsation
    - Percussion: no shifting dulllness, no hepatomegaly, no splenomagaly
  -

Peripheral pulsation :	CA	BA	RA	FA	PA	PTA	DPA
Right side :	++	++	++	++	++	++	++/+++
Left side :	++	++	++	++	++	++	++/+++

  - Genito-anus: no tenderness or palpable mass lesion or rectal shelf, normal external genitalia, normal anal tone, brown stool

CRS CONDITION

- CEA: 2.08 ng/ml
- Imaging studies(Sono , CT of abdomen and chest Films):
  - 檢驗項目 :CT ABDOMEN-SURVEY 報告日期 :1101020
    主 訴：
    Result:Y Finding: Imaging Report Form for Colorectal Carcinoma (AJCC 8th) 1. Imaging Date: 20211015 Imaging Modality, Imaging by CT scan Imaging Protocol: CT scan of abdomen with IV and oral contrast enhancement 2. Tumor location / Size Location: Sigmoid Rectum Size: Measurable: 6.5 cm (largest diameter) 3. Tumor invasion Tumor invades visceral peritoneum or adjacent organs 4. Regional nodal metastasis N1 | N1b Two or three regional lymph nodes are positive. 5. Distant metastasis (In this study) No or Equivocal 6. Other findings: > A patient with Hx of rectal CA s/p tissue proof, for survey. > Circuferential wall thickening with extraluminal invasion and pericolic fat stranding over rectosigmoid colon (Axi 69, Cor27), compatible with rectosigmoid CA. Suggest further management > Multiple smaller than 1 cm lymph node over pelvis region and bilateral iliac chains, suggest clinical correlation. > Some small hepatic cysts > A small poor enhancing nodule 0.6 cm over left adrenal gland, r/o adenoma. Suggest follow-up. > A renal cyst about 1.6cm over right kidney > Mild bilateral hydrocele > The appearance of gallbladder, pancreas, spleen are without significant abnormality. Impression: C/W rectosigmoid CA. Suggest further management
-
  - Lung

手術日期及方法

- 手術日期及方法20211021 1. Robotic (XI) assisted low anterior resection
  2. Intraoperative colonoscopy : (-).
  3. High ligation (+) : At IMA root. Left colic artery and IMV were also ligated
  4. Take down from the splenic flexure : (-)
  5. Robotic endoGIA (45-4.3) X2 + EEA (29mm) were used
  6. Proximal cut end: 5 cm, distal cut end: 5 cm
  7. No drainage tube was inserted
  8. ADHESION BARRIER FILM was placed below the fascia of the central umbilicus wound

住院治療經過

- After admission, operation with robotic LAR was done on 10/21. During operation, a piece of peritoneum was sent for frozen pathology and metastatic adenocarcinoma was proved. We let him try diet under ERAS protocol. Defecation was found on POD1. Under the stable condition, he was discharged on 10/25.

合併症

- nil

一般檢查

- 累積報告：

DATE	TIME	ALB	TP	BIL.T	BIL.D	ALKP	AST	ALT	LDH	AMY	LIPASE	NH3	Ferritin	UA
1101021	1744	3.9		0.87	0.20		18	21						

DATE	TIME	WBC	RBC	HGB	HCT	MCV	PLT	BLST	NEUT	BAND	SEG	LYM	MON	EOS	BAS
1101021	0928	5130	4.62	14.3	42.8	92.6	183		66.8			20.9	5.5	6.2	0.6
1101021	1742	14130		13.7	38.9		158		89.2			6.3	4.3	0.1	0.1
1101021	2254		4.03												

DATE	TIME	NA	K	CL	BUN	CREAT	eGFR	CA	Mg	P	CRP	ESR	PCT	D-Dimer	LACTATE
1101021	1743														14.4
1101021	1744	138	4.4		12	0.97	87.8	8.6							

DATE	TIME	PH	BEB	SCVO2	PO2	HCO3	HGB	PCO2	TCO2
1101021	1743	7.367	-1.6	98.8	146.6	23.7		42.2	25.0

特殊檢查

病理報告

- 活體組織切片病理報告 手術日期：**110/10/21** 病理號：**S1033730**

診斷醫師:文美卿 報告日期:110/10/25
Pathologic diagnosis:
Peritoneum, permanent section of frozen specimen —
Metastatic adenocarcinoma with severe cauterizing artifact. See Note.
Tentative frozen section diagnosis:
Positive for metastatic adenocarcinoma.
Gross description:
The specimen consists of 2 pieces of tan soft tissue, up to 0.1x0.1x0.1 cm, labeled as "peritoneum" for frozen section. The tentative frozen section diagnosis is "Positive for metastatic adenocarcinoma./W". All for section.
Microscopic description:
Section shows fibrotic tissue with infiltrative carcinoma exhibiting glandular differentiation and mucin production. Severe cauterization effect is also seen.
Note:
1. Most of the cancer are lost in the subsequent reprocessed formalin-fixed paraffin embedded tissue.
2. This case has been peer reviewed by two doctors.
#T-D4400_2 #M-81406_2 9 3082 VT000F

其它

出院時情況

- 3.改門診治療

出院指示

藥名	劑量	劑量單位	用法	頻次	數量	數量單位	現況	天數	單位
(口)Takepron OD tab 30mg	1	TAB	PO	QDAC	7	TAB	TH	7	天
Flurbiprofen 100mg	1	TAB	PO	BIDPRN	14	TAB	TH	7	天
Mag. Oxide tab	2	TAB	PO	TID	48	TAB	TH	8	天
Urief tab 4 mg	1	TAB	PO	BID	16	TAB	TH	8	天
BethanechoL tab 25mg-JS	1	TAB	PO	TIDAC	24	TAB	TH	8	天
Acetal tab 500mg	1	TAB	PO	Q6HPRN	28	TAB	TH	7	天

出院計劃

主治醫師 2062F 蔣鋒帆 / 住院醫師 2331F 陳凱祥

正式摘要(電子簽章：**000000228070144)**

W52 / W52

doctorRank:B
